# Silica dust, diesel exhaust, and painting work are the significant occupational risk factors for lung cancer in nonsmoking Chinese men

**DOI:** 10.1038/sj.bjc.6606006

**Published:** 2010-11-23

**Authors:** L A Tse, IT-s Yu, J S K Au, H Qiu, X-r Wang

**Affiliations:** 1 Division of Occupational and Environmental Health, School of Public Health and Primary Care, The Chinese University of Hong Kong, 4/F School of Public Health, Prince of Wales Hospital, Shatin, N.T., HKSAR, China; 2 Department of Clinical Oncology, Queen Elizabeth Hospital, Kowloon, HKSAR, China

**Keywords:** lung carcinoma, nonsmokers, occupation, industry, carcinogen

## Abstract

**Background:**

Few epidemiological studies have explored the associations between occupational exposures and lung cancer in lifelong nonsmoking men.

**Methods:**

We obtained lifetime occupational history and other relevant information for 132 newly diagnosed lung cancer cases among nonsmoking Chinese men and 536 nonsmoking community referents. Unconditional multiple logistic regression analysis was performed to estimate the odds ratio (OR) of lung cancer for specific occupational exposures.

**Results:**

Significantly increased lung cancer risk was found for nonsmoking workers occupationally exposed to silica dust (OR=2.58, 95% confidence interval (CI): 1.11, 6.01), diesel exhaust (OR=3.47, 95% CI: 1.08, 11.14), spray painting (OR=2.81, 95% CI: 1.14, 6.93), and nonspray painting work (OR=2.36, 95% CI: 1.04, 5.37). Silica dust exposure was associated with a significantly increased risk of adenocarcinoma (OR=2.91, 95% CI: 1.10, 7.68). We observed a positive gradient of all lung cancers and of adenocarcinoma with duration of employment for workers exposed to silica dust and spray painting.

**Conclusion:**

This study found an increased risk of lung cancer among nonsmoking Chinese men occupationally exposed to silica dust, diesel exhaust, and painting work.

Tobacco smoking is the most important risk factor for lung cancer (IARC, 2004), contributing around 80% of lung cancer cases of European men and 58% of Chinese men (Tse *et al*, 2009a). Other risk factors include occupational exposures to suspected carcinogens, environmental tobacco smoke (ETS), residential radon exposure, and genetic susceptibility (Alberg *et al*, 2005). Numerous studies have examined the associations between occupational exposures and lung cancer risk, but only a few have explored these in lifelong nonsmoking men (Pohlabeln *et al*, 2000; Zeka *et al*, 2006). Smoking is such a strong risk factor of lung cancer (IARC, 2004) that its presence makes it difficulty to examine the effects of occupational exposures with weak to moderate carcinogenicity. Moreover, cigarette smoking may modify the effects of occupational exposures on lung cancer risk (Liddell, 2001; Yu and Tse, 2006). As inadequate consideration of smoking can lead to inaccurate risk estimations, restriction of study subjects to lifelong nonsmokers offers the best way to examine the independent effects of occupational carcinogens. However, in most studies, lung cancers in lifelong nonsmokers are very few (<10%) to have high statistical power (Peto *et al*, 2000). We used a subgroup of lifelong nonsmokers from a large population-based case–referent study to examine the independent effects of occupational exposures on the risk of all lung cancers combined as well as the histological subtype of adenocarcinoma among Chinese nonsmoking men.

## Materials and methods

The recruitment of cases and referents of this population-based study has been described elsewhere (Tse *et al*, 2009b). In brief, we recruited 1208 newly diagnosed and histologically confirmed primary lung cancer consecutively from the largest oncology centre in Hong Kong from 1 February 2004 to 30 September 2006, with a response rate of 96%. All the eligible cases were Chinese men aged 35–79 years. We recruited 1069 male community referents randomly selected from same districts of the cases, with a response rate of 48%, a response rate comparable with that of other similar studies. Each community referent from the original larger study was frequency matched in 5-year age groups to a lung cancer case, as the age distribution of the subgroup of lifelong nonsmoking cases may not be comparable with the nonsmoking referents. All community referents must have no history of physician-diagnosed cancer in any site. This study was approved by the ethics committees of both the Chinese University of Hong Kong and Queen Elizabeth Hospital.

Personal interviews with the cases and referents were carried out by trained interviewers using structured questionnaires immediately after informed consent was obtained. A lifestyle questionnaire obtained information on demographic data, sources of indoor air pollutants (i.e. exposure to residential radon and ETS, incense burning, use of mosquito coils, and years of cooking by frying), habits of tobacco smoking and alcohol drinking, dietary habits, past history of lung diseases, cancer history in first-degree relatives, and occupational exposures. Histological findings were retrieved from the hospital records. For this study, we selected the subgroup of never smokers defined by subjects who had never smoked as many as 20 packs of cigarettes or 12 oz (342 g) of tobacco in lifetime or one cigarette a day or one cigar a week for 1 year (Ferris, 1978).

A complete work history of jobs held at least 1 year was recorded for each case and referent. The work history included job title, job task description, and the beginning and end date of each job. Job titles and industries were coded according to the International Standard Classification of Occupations (ISCO) and International Standard Industrial Classification of All Economic Activities (ISIC), respectively, for international comparison (International Labour Office, 1968; United Nations Publications, 1971). The whole process of coding was performed blinded to the disease status of subjects.

Additional information on each worker’s regular exposure to specific individual or group of agents from each workplace was captured based on a list of confirmed or suspected human carcinogens, including asbestos, arsenic, nickel, chromium, tars, asphalts, silica, spray painting, nonspray painting, pesticides, diesel engine exhaust, cooking fumes, welding fumes, and man-made mineral fibres. Regular exposure referred to exposure at least once a week for at least 6 months. We introduced the study as a general ‘male health’ study to both cases and referents to minimise the potential recall bias.

We performed unconditional logistic regression models to estimate the odds ratio (OR) and the 95% confidence interval (CI) for lung cancer related to occupations, industries, and exposure to specific or group of agents. In building the model, we initially included various potential confounding factors into a ‘base’ model using a forward stepwise method; the variables that were statistically significant and finally retained in the ‘base’ model were age, place of birth, education level, residential radon exposure, past history of lung diseases, any cancer in first-degree relatives, and intake of meat. We then forced each occupational exposure (i.e. occupations, industries, and exposure to specific or group of agents) into the ‘base’ model to estimate the adjusted OR. Preliminary analyses were performed for major ISCO and ISIC groups; if elevated ORs (ever *vs* never exposed) were suggested, further analyses were conducted in the submajor groups. Two reference groups were applied to each comparison: (1) workers who had never worked in that occupation or industry or had never been exposed to that defined agent (or group of agents) and (2) workers who had never been exposed to any of the confirmed or suspected human carcinogens in the list. Analyses were carried out separately for adenocarcinoma – the most common histological type among nonsmokers (89 cases, 69.4% of all lung cancers).

## Results

This study included 132 nonsmoking male cases and 536 nonsmoking male referents (Table 1). Cases were more likely to be alcohol drinkers, divorced, and exposed to ETS, but they were less educated and had lower family income; a statistical significance was only observed for family income. The mean age of cases was 2.2 years older than the referents (61.9 years) at the time of diagnosis. As described previously, we found that more cases were exposed to higher level of residential radon and had any cancer in first-degree relatives than the referents (Tse *et al*, 2009b).

The ORs of lung cancer for employment in major industries and occupations are presented in Table 2. After adjustment of residential radon exposure and other potential confounding factors, only workers ever employed as ‘bricklayers, carpenters and other construction workers (ISCO code: 9–5)’ showed a significantly increased OR of 2.25 (95% CI: 1.11, 4.54). Elevated ORs were also suggested for some other industries and occupations, but no statistical significance was observed.

The ORs of lung cancer for occupational exposure to specific or group of agents are shown in Table 3. Significantly increased risk was associated with silica dust, painting, and diesel exhaust. Exposure to spray painting showed a 19% higher lung cancer risk than the nonspray painting work. An increased risk of lung cancer was associated with the increasing years of employment for workers exposed to silica dust and spray painting (Table 4).

Separate analyses were repeated for the risk of adenocarcinoma (Tables 3 and 4). We found that a significant OR (2.91, 95% CI: 1.10, 7.68) was retained only for workers exposed to silica dust with an indication of exposure–response relationship with duration of employment. A positive gradient was also observed for painting workers regardless of the process of spray. The risk estimates tended to be stronger when the reference group was replaced by a group of men who had never been exposed to any of the confirmed or suspected human carcinogens in the list (Tables 3 and 4). We further examined the correlation between occupational agents and found no obvious correlation between them (*r*=0.01–0.35). No important effect modification by exposure to ETS was identified for the associations between these defined occupational exposures and lung cancer.

## Discussion

This population-based case–referent study aimed to identify occupational exposures related to elevated risk of lung cancer among lifelong nonsmoking Chinese men in Hong Kong. We found that the groups with employment as ‘bricklayers, carpenters, and other construction workers’ or occupational exposure to silica dust, diesel exhaust, or painting were associated with a significantly increased risk of lung cancer. On account of the small number of subjects in each specific industry or occupation, we analysed risk in major industrial and occupational groups, and found an increased risk of lung cancer among men who had ever been employed as ‘bricklayers, carpenters, and other construction workers’. In Hong Kong, workers employed in construction and the related work accounts for around 9% of the local workforce, while employment as ‘bricklayers, carpenters, and other construction workers’ is the major occupation of local construction industry involving several job tasks, such as stone cutting, pneumatic drilling, caisson, tunnelling, dynamiting, rock sand blasting (already banned), stone crushing, cement machine attendant, bricklayer, decoration work, truck driving or operators of excavating machines, and unskilled labourers (Yu *et al*, 2007). These job tasks are frequently linked to several occupational hazards, in particular, silica dust, diesel exhausts, and painting work. All these occupational exposures are confirmed or suspected as associated with an increased risk of lung cancer (IARC, 1989, 1997, 2009).

Crystalline silica dust has been reclassified as a human group 1 carcinogen by the International Agency for Research on Cancer (IARC) in 1997 (IARC, 1997), while its carcinogenicity has long been debated as potentially confounding the effect of smoking (McDonald and Cherry, 1999; Checkoway and Franzblau, 2000; Hessel *et al*, 2000; Pelucchi *et al*, 2006). We estimated the independent effect of silica dust in nonsmokers, thus avoiding this problem. A recently published multicentre case–referent study in Europe found an OR of 1.76 (95% CI: 0.97, 3.21) in nonsmoking subjects who had ever been exposed to silica dust, and a higher OR in the longest duration of employment group (OR=2.39, 95% CI: 1.11, 5.15, based on 223 cases of which 48 were male) after adjustment of age, sex, and study centre (Zeka *et al*, 2006). The same group of workers found an OR of 1.41 (95% CI: 0.79, 2.49) after redefining the nonexposure group as subjects not exposed to silica dust for >20 years before interview (Cassidy *et al*, 2007). We observed an OR of 2.58 among male workers ever exposed to silica dust and a positive association with increasing years of employment. We carried out a sensitivity analysis and found that the risk estimate was almost unchanged (OR=2.55, 95% CI: 1.14, 5.73) after redefining the nonsmoking status as that of the European study – a man who smoked <100 cigarettes in his lifetime (Zeka *et al*, 2006). We further re-estimated the results by removing ‘any cancer in first-degree relatives’, ‘past history of lung diseases’, and ‘meat intake’ (these variables were not considered in Cassidy’s study) from the model, and found that the OR was reduced by 6.2% (OR=2.42, 95% CI: 1.07, 5.49), but it was still higher than those reported by Zeka *et al* (2006). Our study provides supportive evidence for an independent effect of crystalline silica on lung cancer risk among nonsmokers.

About 18% of our nonsmoking lung cancer cases had been involved in painting work and majority of them were assigned in renovation work of construction or car renewals in which spray painting is frequently required. Employment as a painter has been listed as a human group 1 carcinogen (IARC, 1989); two recent reviews support the conclusion of IARC after assessing reports on painters published since 1951 and a weak association for lung cancer risk (1.22 and 1.36) was shown after controlling for smoking (Bachand *et al*, 2010; Guha *et al*, 2010). Occupational exposure as a spray painter has been associated with an increased risk of urinary tract and testicular cancer, whereas its separate effect on lung cancer has not yet been reported (IARC, 1989). We found a slightly higher risk of lung cancer among painting workers using spray at work (OR=2.81) than general painters whose work never involved spraying (OR=2.36), suggesting exposure differences. Painting workers are commonly exposed by inhalation of solvents and paint dusts (e.g. silica dusts, asbestos dusts, and heavy metals) (IARC, 1989), while spray painting workers may be additionally exposed to a variety of suspected carcinogens in the form of aerosol or fine particles, which can be readily absorbed deep into the lungs (Sabty-daily *et al*, 2005). Our positive association with years of employment as spray painters corroborates the IARC conclusion.

Previous studies showed an average of 33% (95% CI: 24, 44%) excess risk of lung cancer among railroad workers and truck drivers occupationally exposed to diesel engine exhaust emissions, but were commonly criticised for the lack of reliable exposure assessment and inadequate control for smoking. The IARC evaluated diesel exhaust as a group 2A carcinogen because of the limited evidence of carcinogenicity in humans (IARC, 2009). We observed an OR of 3.47 among nonsmoking men occupationally exposed to diesel exhaust, which is much higher than previously reported, but may well be as only six cases of lung cancer were exposed to diesel exhaust and there was no gradient with duration of employment.

There have been few studies of occupation and histological types of lung cancer among lifelong nonsmoking men. Our study numbers allowed us to explore only the risk of adenocarcinoma (the commonest histological type), and among nonsmoking men, we found a slightly higher association with silica dust exposure (a significant OR retained), but a relatively lower OR for occupational exposure to painting and diesel exhaust than all lung cancer cases. The relatively wide CIs of many of risk estimates indicate our limited powers for investigating associations with adenocarcinoma risk, while the multiple comparisons point to the possibility that some significant results have occurred by chance.

Accuracies in recall of nonsmoking status of our subjects (>0.95) and selection bias for the cases and community referents have been addressed in another paper about ETS and lung cancer (Tse *et al*, 2009b). Misclassification of self-reported occupational exposures is a concern because the workers might not accurately identify the specific hazards in their working environments, but these are likely to be nondifferential between cases and referents, resulting in under-estimation of risk. Also, it is difficult to detangle the effects of different job tasks when workers were employed in several occupations during their lifetimes and thus potentially exposed to multiple chemical substances, which indeed may occur even if only one occupation was involved. We are aware that using ‘ever exposure’ might not be a good measurement to quantify the independent effect of an occupational exposure. This study is, therefore, only preliminary and needs confirmation.

To further evaluate the potential recall and/or interviewer bias, we interviewed a subgroup of 45 proxy respondents (e.g. spouse) 2 months after the initial interview and found the overall agreement on occupational exposures was excellent (*κ*=0.72). The test–retest reliability for the same respondents was also very good for both cases (*κ*=0.65) and referents (*κ*=0.60). We further interviewed a special group of 64 inpatient referents (who had to undergo surgical operations for suspected lung cancer and were treated as lung cancer cases at the interviews, but eventually were diagnosed as not suffering from lung cancer) who showed a lower proportion of occupational exposures than the confirmed lung cancer cases, suggesting that any interviewer or recall bias was not a major issue.

Our study found that men employed as ‘bricklayers, carpenters, and other construction workers’ and those who had ever been occupationally exposed to silica dust, diesel exhaust, and painting work were associated with an increased risk of all lung cancers, and the effects were independent of smoking.

## Figures and Tables

**Table 1:** Selected characteristics of lung cancer cases and community referents in nonsmoking Chinese men during 2004–2006

	**Cases**		**Community referents**		
**Characteristics by category**	**No.**	**%**	**No.**	**%**	***P-*value^a^**
Total no.	132		536		
					
*Age at interview (year)*					0.038
<50	22	16.7	63	11.8	
50–59	36	27.3	108	20.1	
60–69	40	30.3	166	31.0	
⩾70	34	25.8	199	37.1	
					
*Family monthly income (HK dollar)* b					0.001
<4000	55	41.7	202	37.7	
⩾4000	54	40.9	157	29.3	
Not answered	23	17.4	177	33.0	
					
*Marital status*					0.351
Single	8	6.1	38	7.1	
Married	115	87.1	450	84.0	
Widowed	2	1.5	23	4.3	
Divorced	7	5.3	19	3.5	
					
*Education level*					0.681
Illiterate	14	10.6	54	10.1	
Primary	47	35.6	163	30.4	
High school	46	34.8	201	37.5	
College or above	24	18.2	107	20.0	
					
*ETS in workplace or at home*					0.613
Never	42	31.8	183	34.1	
Ever	90	68.2	353	65.9	
					
*Alcohol drinking* c					0.120
Never	69	52.3	333	62.1	
Occasional	34	25.8	110	20.5	
Frequent	28	21.2	90	16.8	

Abbreviations: ETS=environmental tobacco smoke; HK=Hong Kong.

a *P-*value from Person’s *χ*^2^ test.

b 1 US dollar=7.8 HK dollar.

c Occasional drinkers referred to those who drank at most three times per week and frequent drinkers referred to those who drank four times or more per week.

**Table 2:** OR and 95% confidence intervals for lung cancer by major groups of industry and occupation in nonsmoking Chinese men^a^^,^^b^

		**Referents (*n*=536)**	**All lung cancers (*n*=132)**			**Adenocarcinoma (*n*=89)**		
**ISIC/ISCO codes**	**Category**	***n* (%)**	***n* (%)**	**Crude OR**	**Adjusted OR^c^**	***n* (%)**	**Crude OR**	**Adjusted OR^c^**
*ISIC*	*Major industrial division*							
1	Agriculture, hunting, forestry, and fishing	1 (0.2)	1 (0.8)	4.08 (0.25, 65.72)	7.36 (0.28, 190.77)	1 (1.1)	6.08 (0.38, 98.09)	19.29 (0.72, 502.49)
3	Manufacturing	98 (18.3)	28 (21.2)	1.20 (0.75, 1.93)	1.00 (0.58, 1.70)	18 (20.2)	1.13 (0.65, 1.99)	0.95 (0.51, 1.79)
5	Construction	37 (6.9)	15 (11.4)	1.73 (0.92, 3.26)	1.61 (0.79, 3.26)	10 (11.2)	1.71 (0.82, 3.57)	1.68 (0.74, 3.84)
6	Wholesale and retail trade, and restaurants and hotels	78 (14.6)	18 (13.6)	0.93 (0.53, 1.61)	1.04 (0.55, 1.94)	12 (13.5)	0.92 (0.48, 1.76)	1.00 (0.48, 2.10)
7	Transport, storage, and communication	80 (14.9)	16 (12.1)	0.79 (0.44, 1.40)	0.71 (0.37, 1.35)	9 (10.1)	0.64 (0.31, 1.33)	0.60 (0.27, 1.33)
8	Financing, insurance, real estate, and business services	34 (6.3)	3 (2.3)	0.34 (0.10, 1.14)	0.43 (0.12, 1.55)	2 (2.2)	0.34 (0.08, 1.44)	0.40 (0.09, 1.85)
9	Community, social, and personal services	149 (27.8)	35 (26.5)	0.94 (0.61, 1.44)	1.02 (0.62, 1.67)	25 (28.1)	1.02 (0.62, 1.67)	0.97 (0.55, 1.69)
10	Activities not adequately defined	34 (6.3)	8 (6.1)	0.95 (0.43, 2.11)	1.02 (0.41, 2.53)	6 (6.7)	1.07 (0.44, 2.62)	1.21 (0.45, 3.26)
								
*ISCO*	*Major occupational group*							
0/1	Professional, technical, and related workers	50 (9.3)	14 (10.6)	1.15 (0.62, 2.16)	1.58 (0.76, 3.29)	7 (7.9)	0.83 (0.36, 1.89)	0.90 (0.35, 2.29)
2	Administrative and managerial workers	29 (5.4)	7 (5.3)	0.98 (0.42, 2.29)	1.18 (0.47, 2.97)	5 (5.6)	1.04 (0.39, 2.76)	1.19 (0.42, 3.36)
3	Clerical and related workers	47 (8.8)	16 (12.1)	1.44 (0.79, 2.62)	1.53 (0.76, 3.08)	11 (12.4)	1.47 (0.73, 2.95)	1.48 (0.67, 3.29)
4	Sales workers	77 (14.4)	15 (11.4)	0.76 (0.42, 1.38)	0.79 (0.41, 1.54)	10 (11.2)	0.76 (0.37, 1.52)	0.65 (0.29, 1.46)
5	Services	111 (20.7)	30 (22.7)	1.13 (0.71, 1.78)	0.90 (0.53, 1.53)	23 (25.8)	1.33 (0.79, 2.24)	1.09 (0.60, 1.98)
6	Agricultural animal husbandry and forestry workers, fishermen, and hunters	16 (3.0)	3 (2.3)	0.76 (0.22, 2.63)	0.78 (0.20, 2.97)	3 (3.4)	1.13 (0.32, 3.97)	1.21 (0.31, 4.68)
7/8/9	Production and related workers, transport equipment operators and labourers	285 (53.2)	87 (65.9)	**1.70 (1.14, 2.54)**	1.58 (0.99, 2.54)	61 (68.5)	**1.92 (1.19, 3.10)**	**1.72 (1.00, 2.98)**
7–0	Production supervisors and general foremen	74 (13.8)	21 (15.9)	1.18 (0.70, 2.00)	0.88 (0.48, 1.62)	12 (13.5)	0.97 (0.51, 1.88)	0.68 (0.32, 1.46)
8–0	Shoemaker and leather goods makers	86 (16.0)	27 (20.5)	1.35 (0.83, 2.18)	1.41 (0.82, 2.42)	19 (21.3)	1.42 (0.81, 2.48)	1.57 (0.85, 2.90)
9–5	Bricklayers, carpenters, and other construction workers	32 (6.0)	16 (12.1)	**2.17 (1.15, 4.09)**	**2.25 (1.11, 4.54)**	10 (11.2)	1.99 (0.94, 4.22)	2.11 (0.94, 4.75)

Abbreviations: OR=odds ratio; ISIC=International Standard Industrial Classification of All Economic Activities; ISCO=International Standard Classification of Occupations.

^a^The reference group consists of never employed in given industries or occupations.

^b^There is no case in the category of ISIC 2 and 4.

c Models were adjusted for age, place of birth, education level, residential radon exposure, past history of lung diseases, any cancer in first-degree relatives, and intake of meat. Bold values indicate *P*<0.05.

**Table 3:** OR of lung cancer and 95% confidence intervals by occupational exposure to the individual specific or group of agents^a^

	**Referents (*n*=536)**	**Lung cancer cases (*n*=132)**			**Adenocarcinoma (*n*=89)**		
**Occupational exposures**	***n* (%)**	***n* (%)**	**Adjusted OR_1_^b^**	**Adjusted OR_2_^c^**	***n* (%)**	**Adjusted OR_1_^b^**	**Adjusted OR_2_^c^**
Asbestos dust	23 (4.3)	4 (3.0)	0.77 (0.23, 2.55)	0.99 (0.30, 3.32)	3 (3.4)	0.98 (0.27, 3.57)	1.23 (0.33, 4.58)
Silica dust	20 (3.7)	10 (7.6)	**2.58 (1.11, 6.01)**	**3.09 (1.30, 7.37)**	7 (7.9)	**2.91 (1.10, 7.68)**	**3.41 (1.26, 9.25)**
General painting work not involving spray	25 (4.7)	12 (9.1)	**2.36 (1.04, 5.37)**	**2.79 (1.20, 6.48)**	7 (7.9)	2.07 (0.79, 5.47)	2.48 (0.92, 6.70)
Spray painting work	20 (3.7)	11 (8.3)	**2.81 (1.14, 6.93)**	**3.29 (1.31, 8.23)**	7 (7.9)	2.62 (0.91, 7.56)	**3.03 (1.03, 8.92)**
Welding fume	18 (3.4)	9 (6.8)	1.81 (0.73, 4.49)	2.24 (0.88, 5.69)	6 (6.7)	1.72 (0.59, 5.00)	2.11 (0.71, 6.29)
Emissions from plastic heating or melting	3 (0.6)	2 (1.5)	3.47 (0.42, 28.85)	4.49 (0.54, 37.69)	1 (1.1)	2.99 (0.22, 40.39)	3.61 (0.27, 49.05)
Chromium	5 (0.9)	1 (0.8)	1.38 (0.14, 13.83)	1.72 (0.17, 17.38)	1 (1.1)	1.64 (0.15, 17.78)	2.09 (0.19, 22.70)
Pesticide users	8 (1.5)	3 (2.3)	1.07 (0.20, 5.73)	1.32 (0.25, 7.13)	3 (3.4)	1.14 (0.21, 6.25)	1.45 (0.26, 8.05)
Diesel exhaust	13 (2.4)	6 (4.5)	**3.47 (1.08, 11.14)**	**4.17 (1.28, 13.53)**	3 (3.4)	1.92 (0.44, 8.39)	2.38 (0.54, 10.60)
Gasoline exhaust	16 (3.0)	7 (5.3)	1.21 (0.42, 3.56)	1.52 (0.51, 4.53)	4 (4.5)	1.05 (0.26, 4.20)	1.29 (0.32, 5.27)
Cooking fume	23 (4.3)	6 (4.5)	0.77 (0.27, 2.25)	0.98 (0.33, 2.91)	4 (4.5)	0.95 (0.29, 3.10)	1.18 (0.36, 3.94)
Rubber handling	6 (1.1)	2 (1.5)	1.47 (0.27, 8.05)	1.82 (0.33, 10.11)	1 (1.1)	0.76 (0.08, 7.19)	0.99 (0.10, 9.49)
Organic solvent	84 (15.7)	22 (16.7)	0.96 (0.54, 1.73)	1.24 (0.67, 2.29)	13 (14.6)	0.85 (0.42, 1.73)	1.10 (0.52, 2.30)
Wood dust	7 (1.3)	2 (1.5)	1.41 (0.26, 7.59)	1.79 (0.33, 9.75)	2 (2.2)	2.16 (0.39, 11.98)	2.68 (0.48, 15.03)
Textile dust	56 (10.4)	12 (9.1)	0.56 (0.26, 1.19)	0.76 (0.34, 1.66)	9 (10.1)	0.56 (0.23, 1.35)	0.75 (0.30, 1.87)
Man-made mineral fibre	3 (0.6)	2 (1.5)	4.63 (0.71, 30.02)	5.74 (0.87, 37.71)	1 (1.1)	3.10 (0.30, 32.46)	3.85 (0.37, 40.66)

Abbreviation: OR=odds ratio.

a Models were adjusted for age, place of birth, education level, residential radon exposure, past history of lung diseases, any cancer in first-degree relatives, and intake of meat.

b The reference group consists of never exposed to the specific agent.

c The reference group consists of never exposed to any of the confirmed or suspected human carcinogens in the list. Bold values indicate *P*<0.05.

**Table 4:** OR of lung cancer and the 95% confidence intervals for occupational exposures to selected individual specific or group of agents among nonsmoking men^a^

	**Referents (*n*=536)**	**Lung cancer cases (*n*=132)**			**Adenocarcinoma (*n*=89)**		
**Years of exposure**	***n* (%)**	***n* (%)**	**Adjusted OR_1_^b^**	**Adjusted OR_2_^c^**	***n* (%)**	**Adjusted OR_1_^b^**	**Adjusted OR_2_^c^**
*Silica dust*							
Reference 1^b^	516 (96.3)	122 (92.4)	1.00	—	82 (92.1)	1.00	—
Reference 2^c^	344 (64.2)	61 (46.2)	—	1.00	41 (46.1)	—	1.00
1–19 years	11 (2.1)	5 (3.8)	2.23 (0.73, 6.81)	2.68 (0.86, 8.35)	3 (3.4)	1.99 (0.51, 7.74)	2.35 (0.59, 9.31)
⩾20 years	9 (1.7)	5 (3.8)	3.13 (0.91, 10.79)	**3.72** **(1.06, 13.00)**	4 (4.5)	4.50 **(1.17, 17.33)**	**5.23 (1.34, 20.44)**
							
*General painting work not involving spray*							
Reference 1^b^	511 (95.3)	120 (90.9)	1.00	—	82 (92.1)	1.00	—
Reference 2^c^	344 (64.2)	61 (46.2)	—	1.00	41 (46.1)	—	1.00
1–19 years	10 (1.9)	5 (3.8)	2.37 (0.68, 8.24)	2.80 (0.80, 9.86)	3 (3.4)	1.90 (0.42, 8.57)	2.29 (0.50, 10.42)
⩾20 years	15 (2.8)	7 (5.3)	2.36 (0.83, 6.72)	2.79 (0.96, 8.08)	4 (4.5)	2.20 (0.65, 7.47)	2.61 (0.75, 9.05)
							
*Spray painting work*							
Reference 1^b^	516 (96.3)	121 (91.7)	1.00	—	82 (92.1)	1.00	—
Reference 2^c^	344 (64.2)	61 (46.2)	—	1.00	41 (46.1)	—	1.00
1–19 years	7 (1.3)	4 (3.0)	1.59 (0.32, 7.96)	1.86 (0.37, 9.34)	3 (3.4)	1.56 (0.27, 9.12)	1.83 (0.31, 10.82)
⩾20 years	13 (2.4)	7 (5.3)	**3.66 (1.26, 10.59)**	**4.28 (1.46, 12.57)**	4 (4.5)	3.56 (0.99, 12.79)	4.08 (1.12, 14.84)
							
*Diesel exhaust*							
Reference 1^b^	523 (97.6)	126 (95.5)	1.00	—	86 (96.6)	1.00	—
Reference 2^c^	344 (64.2)	61 (46.2)	—	1.00	41 (46.1)	—	1.00
1–19 years	4 (0.7)	4 (3.0)	**12.39 (2.40, 63.94)**	**14.69 (2.83, 76.32)**	2 (2.2)	6.16 (0.77, 49.27)	7.56 (0.94, 60.83)
⩾20 years	9 (1.7)	2 (1.5)	1.05 (0.18, 6.26)	1.28 (0.22, 7.65)	1 (1.1)	0.75 (0.08, 7.36)	0.94 (0.10, 9.28)

Abbreviation: OR=odds ratio.

a Models were adjusted for age, place of birth, education level, residential radon exposure, past history of lung diseases, any cancer in first-degree relatives, and intake of meat.

b The reference group consists of never exposed to the specific agent.

c The reference group consists of never exposed to any of the confirmed or suspected human carcinogens in the list. Bold values indicate *P*<0.05.
